# Association between tumor necrosis factor α and uterine fibroids: A protocol of systematic review

**DOI:** 10.1097/MD.0000000000021667

**Published:** 2020-08-14

**Authors:** Li-nan Gao, Lian-gang Ge, Ming-zhe Zhu, Xin-xin Yao

**Affiliations:** a Department of Laboratory, Affiliated Hospital of Jilin Medical University; b Department of Radiotherapy, The Second People's Hospital of Jilin; c Department of Obstetrics and Gynecology; d Department of Pathology, Affiliated Hospital of Jilin Medical University, Jilin, Jilin Province, China.

**Keywords:** association, tumor necrosis factor α, uterine fibroids

## Abstract

**Background:**

This study will explore the association between tumor necrosis factor α (TNF-α) and uterine fibroids (UFs).

**Methods:**

We will retrieve electronic databases in Cochrane Library, PUBMED, EMBASE, Web of Science, WANGFANG, Chinese Biomedical Literature Database, and China National Knowledge Infrastructure from inception to the present. All potential case-controlled studies investigating the association between TNF-α and UFs will be included in this study. Two researchers will independently select literature, appraise study quality, and extract outcome data. We will utilize a fixed-effects model or a random-effects model to synthesize outcome data. All data analysis will be performed by RevMan 5.3 software.

**Results:**

The present study will supply high-quality synthesis and/or descriptive analysis of the recent evidence to explore the association between TNF-α and UFs.

**Conclusion:**

This study will exert evidence to determine whether or not TNF-α is associated with UFs.

**Study registration number:**

INPLASY202070010.

## 1 Introduction

Uterine fibroids (UFs), also known as leiomyomas, are among the most common benign pelvic tumors in females of reproductive years.^[1–3]^ It manifests as heavy menstrual bleeding, menstrual periods lasting over a week, pelvic pressure or pain, frequent urination, and difficulty emptying the bladder.^[4–7]^ It has been reported that its incidence is directly associated to the age, varying from 40% to 60% at 35 years old to 70% to 80% at 50 years old.^[7–11]^ Its prevalence ranges from 0.1% to 10.7% in pregnant women.^[12–13]^ Several risk factors maybe responsible for UFs, including genetic changes, hormones, extracellular matrix, and other growth factors, such as tumor necrosis factor α (TNF-α), that may affect UFs growth.^[14–19]^

A variety of studies reported that TNF-α is associated with UFs.^[20–24]^ However, there is no systematic review exploring the association between TNF-α and UFs.^[20–24]^ Therefore, with a growing number of studies focusing on this topic, the present study will systematically appraise the association between TNF-α and UFs.

## 2 Methods

### 2.1 Study registration

This study was registered on INPLASY202070010. It has been organized following the guideline of Preferred Reporting Items for Systematic Reviews and Meta-Analysis Protocol statement.^[25]^

### 2.2 Criteria for included studies

All potential case-controlled studies exploring the association between TNF-α and UFs will be considered.

Patients who were diagnosed as UFs will be included in the experimental group, and normal healthy participants will be considered in the control group, in spite of country, race, and age.

We will assess the outcome indicators based on the studies concerning the association between TNF-α and UFs, such as gene and protein expression of TNF-α, proportion requiring hysterectomy, quality of life, and successful pregnancies.

### 2.3 Strategy of literature searches

From inception to the present, electronic databases will be searched in Cochrane Library, PUBMED, EMBASE, Web of Science, WANGFANG, Chinese Biomedical Literature Database, and China National Knowledge Infrastructure. We will consider case-controlled studies addressing the association between TNF-α and UFs. The template of search strategy of PUBMED is summarized in Table 1. Identical search strategies for other electronic databases will be modified.

**Table 1:**
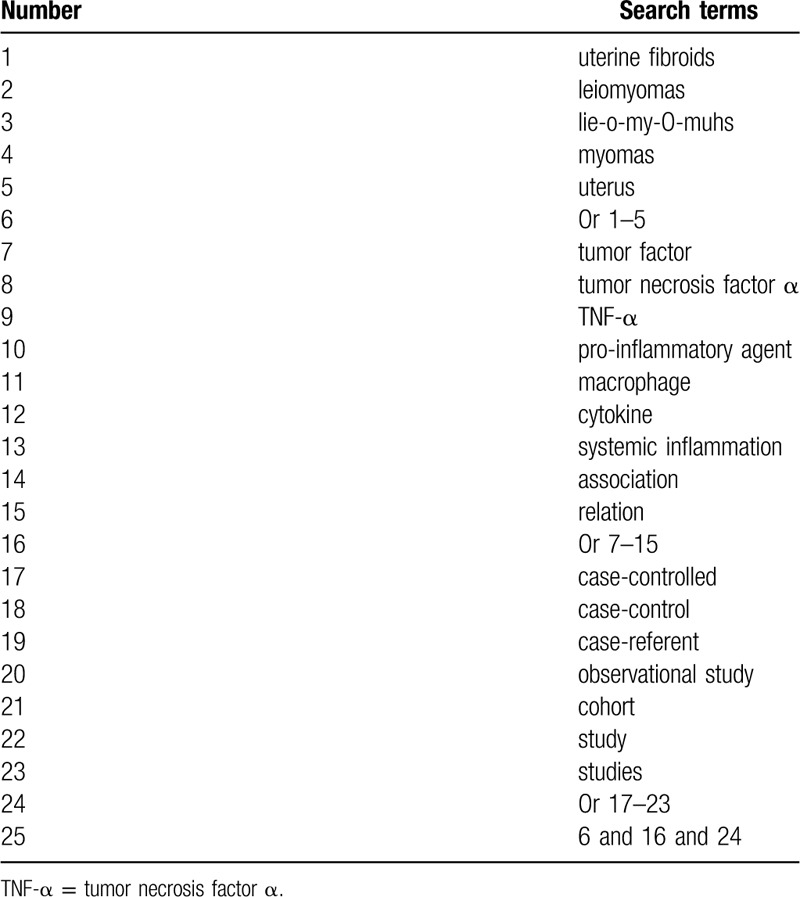
Detailed search strategy of PUBMED.

In addition, we will search ongoing studies in clinical registry trials, conference proceedings, and reference lists of relevant reviews.

### 2.4 Data collection

#### 2.4.1 Study selection

Two researchers will export all searched records to Endnote Software (X9); and repetitive studies will be eliminated. After getting rid of the duplications, titles/abstracts for potentially qualified studies will be scanned to remove irrelevant ones. Then, we will check full-text of potential studies against all eligibility criteria. If inconsistent opinions occur, we will solve it though discussion by a third researcher. We will supply the process and results of study selection in a flow chart. We will unravel any disparity by discussion with the help of another researcher.

#### 2.4.2 Data collection

Two researchers will independently collect data using standard data extraction form. The following information consists of basic information (study ID, publication time and source, first author, etc), characteristics of study (study setting, study methods, sample size, etc), intervention and control indexes, outcomes, following up information, results and findings. Any disagreement will be solved by discussion with another researcher.

#### 2.4.3 Dealing with missing data

Any missing information will be obtained from primary trial authors by email or phone. If we can not get such data, we will perform a narrative synthesis of available data.

### 2.5 Study quality assessment

The quality of eligible studies will be assessed by 2 independent researchers using The Newcastle-Ottawa Scale. Any division will be solved by another researcher through consultation, and a consensus will be reached.

### 2.6 Statistical analysis

We will perform RevMan 5.3 software to conduct statistical analysis. The weighted mean difference or standardized mean difference and 95% confidence intervals, and risk ratio and 95% confidence intervals will be estimated to present data synthesis outcome of continuous data and dichotomous data, respectively. Statistical heterogeneity will be checked by *I*^2^ test, and a coarse guide for its explanation is as follows: *I*^2^ < 40% indicates that there might be minor heterogeneity, and we will use a fixed-effects model; 40%≤ *I*^2^ < 75% means moderate heterogeneity; and we will employ a random-effects model; and *I*^2^≥75% means significant heterogeneity, and meta-analysis is deemed not to be performed. If *I*^2^ ≥40%, the source of heterogeneity will be explored using subgroup analysis and meta-regression test.

### 2.7 Additional analysis

Subgroup analysis and meta-regression test will be conducted according to the characteristics of the study participants, study quality, and sample size.

Sensitivity analysis will be performed to examine the robustness of study findings by taking away low study quality.

Reporting bias will be performed by funnel plot^[26]^ and Egger regression test^[27]^ if over 10 eligible studies are included.

### 2.8 Ethics and dissemination

This study will not utilize individual patient data, thus no ethic approval is requested. This study will be published on a peer-reviewed journal.

## 3 Discussion

UFs are very common benign gynecological tumors of reproductive age.^[1–3]^ Many factors are reported to have association with UFs, such as TNF-α.^[14–19]^ Many previous studies reported the association between TNF-α and UFs.^[20–24]^ However, no systematic review has investigated this issue. Thus, this systematic review will explore the association between TNF-α and UFs. We expect that the results of this study may provide beneficial evidence for clinical practice and future studies.

## Author contributions

**Conceptualization:** Li-nan Gao, Ming-zhe Zhu, Xin-xin Yao.

**Data curation:** Li-nan Gao, Ming-zhe Zhu.

**Formal analysis:** Li-nan Gao, Lian-gang Ge.

**Investigation:** Li-nan Gao.

**Methodology:** Ming-zhe Zhu, Xin-xin Yao.

**Project administration:** Li-nan Gao.

**Resources:** Lian-gang Ge, Ming-zhe Zhu, Xin-xin Yao.

**Software:** Lian-gang Ge, Ming-zhe Zhu, Xin-xin Yao.

**Supervision:** Li-nan Gao.

**Validation:** Li-nan Gao, Lian-gang Ge, Ming-zhe Zhu, Xin-xin Yao.

**Visualization:** Li-nan Gao, Lian-gang Ge, Ming-zhe Zhu, Xin-xin Yao.

**Writing – original draft:** Li-nan Gao, Lian-gang Ge.

**Writing – review & editing:** Li-nan Gao, Lian-gang Ge, Ming-zhe Zhu, Xin-xin Yao.
